# Impact of a lifestyle intervention program on cardio-metabolic parameters among obese adults: A comparative population-based study in West Bengal, India

**DOI:** 10.25122/jml-2022-0006

**Published:** 2023-04

**Authors:** Chaitali Bose, Amol Dilip Kinge, Julekha Sultana, Ajoy Kumar Biswas, Koushik Bhattacharya, Alak Kumar Syamal

**Affiliations:** 1 Post-Graduate Department of Physiology, Hooghly Mohsin College, University of Burdwan, Hooghly, West-Bengal, India; 2 Department of Community Medicine, Government Medical College, Nandurbar, Maharastra, India; 3 Department of Medicine, G.D Hospital and Diabetes Institute Kolkata, Kolkata, West-Bengal, India; 4 Department of Physiotherapy, School of Allied Health Sciences, Swami Vivekananda University, Barrackpore, West-Bengal, India

**Keywords:** cardio-metabolic risks, dietary modification, lifestyle, non-communicable diseases, obesity, physical activity, BMI – Body Mass Index, DBP – Diastolic Blood Pressure, FBG – Fasting Blood Glucose, HDL-C – High Density Lipo-protein Cholesterol, HOMA-IR – Homeostatic Model Assessment for Insulin Resistance, LDL-C – Low Density Lipo-protein Cholesterol, MET – Metabolic Equivalent, NCDs – Non-Communicable Diseases, N.S. – Non-Significant i.e. p>0.05, PA – Physical Activity, SBP – Systolic Blood Pressure, SD – Standard Deviation, TC – Total Cholesterol, TG – Triglyceride, WC – Waist Circumference, WHR – Waist to Hip Ratio, WHtR – Waist to Height Ratio

## Abstract

The obesity epidemic is not only limited to high-income or urbanized societies, but has also become prevalent among rural communities, even in India. Approaches to modifiable behaviors, like unhealthy dietary habits or a sedentary lifestyle, could bring positive results among obese populations. This research aimed to assess the effectiveness of lifestyle intervention programs to prevent obesity and cardio-metabolic risks among Bengali obese adults (Body Mass Index of 25-30kg/m^2^). The population was selected from rural and urban communities of Hooghly district in west Bengal, India and included 121 participants (20-50 years), divided into four groups (rural male, rural female, urban male, and urban female) who underwent a 12-month intervention program. Anthropometric parameters, systolic and diastolic blood pressure, biochemical parameters (fasting blood glucose, fasting plasma insulin, Homeostatic Model Assessment for Insulin Resistance [HOMA-IR] and lipid profile), dietary habits, and physical activity profiles were assessed before the study (baseline), after 12 months of intervention (post-intervention), and after 24 months (follow-up), among all groups, to evaluate changes in data within and between the groups (rural vs. urban). The results showed a significant decline in anthropometric parameters and fasting blood glucose levels among all intervention groups, HOMA-IR in rural females, and serum triglyceride levels in urban groups. A significant improvement was noted regarding dietary habits and physical activity, even during follow-up. The impact of the intervention program did not show any rural-urban difference. The lifestyle intervention program was effective in reducing obesity and related health risks and promoting a healthy lifestyle among the target population.

## INTRODUCTION

Obesity can be defined as an accumulation of excess adipose tissue in the body resulting in health risks. The obesity surge has become prominent since 1975 across the world. According to the World Health Organization (WHO), in 2016 nearly 1.9 billion adults were overweight, and 650 million among them were obese. This global epidemic was not only confined to high-income countries, but also sprawling in low- or middle-income countries like India. Research revealed that among such countries, obesity prevalence is increasing at a similar or faster pace in rural areas compared to urban settings [1]. Obesity is a major risk for non-communicable diseases (NCDs) as it substantially raises the threat of metabolic, cardiovascular, respiratory, musculoskeletal, and mental health disorders, as well as for different forms of cancer [2]. Certain NCDs such as cardiovascular diseases, diabetes, and different cancers account for >70% of global deaths yearly and are the leading causes of premature mortality and disabilities [3]. In 2011, the United Nations general assembly recognized unhealthy diet and physical inactivity as key factors to be modified for the prevention and control of NCDs. Obesity, which is a salient risk for NCDs, results from the complex interplay among one’s genetic or epigenetic factors, environment (including intra-uterine environment), and behavior (faulty dietary habits and sedentary living) [4]. Even though the fundamental reason behind obesity is the long-term energy imbalance, a high intake of calories with low expenditure leads to a positive energy balance [5]. WHO also remarked that high-calorie dense food, rich in fat or sugar, a sedentary living promoted by urbanization, and developing social, economic, industrial, agricultural, health, or marketing sectors foster lifestyle transitions that may lead to lifestyle diseases or NCDs [6].

In response to the alarming rate and severity of NCDs globally, the WHO Global Strategy on Diet, Physical Activity and Health 2004 fostered the promotion of healthy living structured around healthy diet and physical activity and maintaining energy balance, as the key factor to prevent obesity. The management of obesity needs a ‘two-pronged’ initiative to (1) provide better surgical, pharmacological, and behavioral interventions, and (2) prevent obesity by tackling environmental factors [7].

Among the interventional measures, behavioral or lifestyle management programs have been gaining ground globally over the last few decades. Many research teams have undertaken such programs to address lifestyle diseases like obesity, diabetes, or other cardio-metabolic disorders [8-9]. These initiatives are mainly based on health or nutrition education and group or individual counseling to adopt modified dietary habits and increased physical activity (PA). The duration of programs varied from three months to two years or more and was supervised by medical practitioners with or without other paramedical staff (e.g., nutritionists, dieticians, nurses, or trained physiotherapists). The Diabetes Prevention Program (DPP) in the USA in 2002 successfully reverted pre-diabetic patients to patients with normal blood glucose levels [10]. In India, the Kerala-Diabetes Prevention Program (K-DPP), a clustered randomized controlled trial, was implemented and resulted in significant improvements regarding cardiovascular risks [11]. Other interventional studies based on lifestyle modification for several NCDs have been implemented in various age groups at different places (schools, outdoor hospitals, clinics, and workplaces), with positive results [12-13]. Studies from West Bengal on the Bengalee population have shown their increasing risks regarding the anthropometric, cardiovascular, and metabolic profile, also accompanied by excess intake of calories and their sedentary lifestyle, both in rural and urban areas, especially among upper-class women [14-15]. No such research was available in West Bengal, so it is the first-ever attempt to administer a lifestyle intervention program targeting an approach to obesity and other cardio-metabolic parameters through dietary modification and increased PA recommendation in both the rural and urban populations.

## MATERIAL AND METHODS

### Research design and framework

This study implemented a longitudinal research design. 121 subjects selected from rural and urban backgrounds of the Hooghly district (age group 20-50 years; both male and female; Body Mass Index of 25-30 kg/m^2^) underwent a lifestyle intervention program for 12 months. The study included measurements of anthropometric characteristics [body weight, height, BMI, Waist Circumference (WC), Waist to Hip Ratio (WHR), Waist to Height Ratio (WHtR)], blood pressure (systolic and diastolic), and biochemical parameters [fasting blood glucose (FBG), fasting insulin, Homeostatic Model Assessment for Insulin Resistance (HOMA-IR), lipid profile] at baseline (before the commencement of the lifestyle program), post-intervention (at 12 months), and at follow-up, which was after another 12 months (i.e., at 24 months). The study was conducted from September 2017 to September 2019. Participants received lifestyle modification and awareness training regarding obesity and related lifestyle diseases, their risks of having various NCDs, the need for weight reduction, and the importance of dietary changes and increased physical exercise. Self-reported dietary behavior and PA levels were checked at regular intervals. Subjects were encouraged by the research team to adopt modified health behaviour through small group sessions at definite intervals.

### Study setting

At first, general data, anthropometric parameters, resting blood pressure (both systolic and diastolic), and dietary and physical activity profiles were collected from the urban and rural areas by a home visit. After screening, the eligible subjects were invited to attend mobile clinics, which were community buildings (school buildings or mobile health check-up camps) and were asked to fast for 8-12 hours before a blood test. Participants with a fasting blood glucose ≥126 mg/dl were rejected for the intervention program as per the study criteria and suggested to visit government health clinics. The intervention program, including data collection, counselling sessions, and meeting with participants, was conducted in community buildings on Saturdays or Sundays, at the convenience of the participants. The study settings had the necessary equipment and spaces ensured by the local volunteers (club members, non-teaching staff, students) along with the research team consisting of trained dieticians, nutritionists, health professionals, lab technicians, and physiologists.

### Eligibility and Recruitment of subjects

Eligible participants were recruited from rural and urban areas of the Hooghly district. Urban areas were municipal areas, and rural areas were the villages under the gram panchayat of Community Development Blocks of Hooghly. The locations were purposively selected depending on accessibility, distance, or familiarity of the research team. After selecting municipal areas wards, the streets were chosen randomly. In rural areas, the process was similar. The households were selected by systematic random sampling, and the subjects were recruited based on study criteria.

### Inclusion criteria

Subjects were Bengali-speaking Hindus, permanent residents of the selected areas, male or female, aged 20-50 years, had at least upper primary or high-school education (8th grade to 12th grade), a BMI of 25-30 kg/m^2^, were free from any clinically diagnosed diseases, were not consuming alcohol or any form of tobacco, had a diet with excess of calories, sugar, or refined carbohydrates, saturated and trans fats, and high in sodium, and were physically inactive (Metabolic Equivalent or MET score <600).

### Exclusion criteria

The research excluded pregnant or lactating women or those planning for conception, people on medication (e.g., steroids, anti-depressant, anti-hypertensive drugs), participants on weight reduction therapy or who have undergone surgery, non-ambulatory participants, or those with physical or mental disabilities, people with eating disorders and those who were diagnosed with certain medical problems before or during the study period (e.g., hypertension, diabetes, stroke) and were not willing to participate [16].

### Sample size

**Figure 1:**
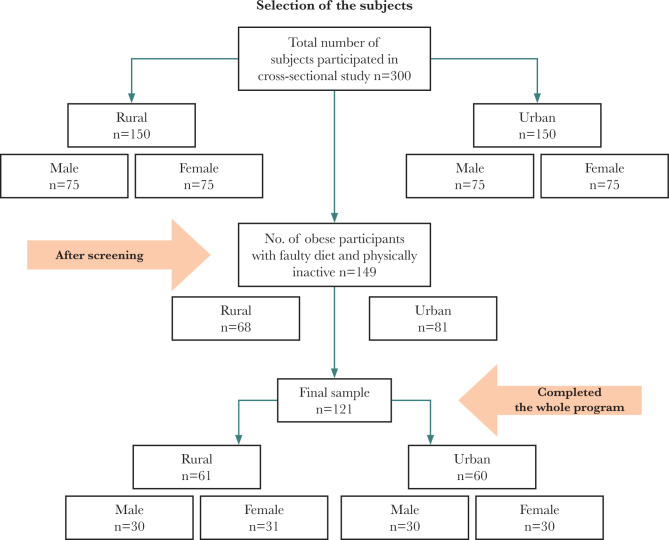
Selection of subjects for the intervention study

A total of 300 subjects with a BMI of 25-30 kg/m^2^ (150 from rural areas, 75 male and 75 female; 150 from the urban area, 75 male and 75 female) were screened before undergoing the intervention program. Obese subjects with a MET score >600 whose diets were not faulty or deficient with macronutrients and were excluded. Subjects matched based on study criteria and with similar socio-demographic backgrounds and occupations from both rural and urban areas were recruited. Out of 300 subjects, 190 (107 in urban areas [Male=48 & Female=59] and 83 in rural areas [Male=39 & Female=44]) were selected. Out of the 190 participants, 41 were excluded after the blood test; thus, the program was started with 149 people (81 from urban areas, 68 from rural areas). A total of 121 (61 from rural area, 60 from the urban area) completed the lifestyle modification program and participated in the follow-up at 12 months and at 24 months. Therefore, we considered 121 participants as the sample size for this study. The sample selection is represented in Figure 1.

### Intervention period

The intervention program was initiated in September 2017 and continued for 12 months. After the follow-up, at 24 months from the initiation (September 2019), the program ended.

### Program activities and content

The activities were based on previous interventions for reducing obesity, diabetes, or cardiovascular risks through lifestyle modification in India or other countries for adults with metabolic risks [17-19].

The program included: the inaugural session, individual and group-based counseling sessions for dietary modification, PA recommendations, behavioral modification, and sessions on motivation, problem-solving, and encouragement to adaptation. The tracked variables included participants' self-recorded data, anthropometric parameters, blood pressure, and biochemical parameters, assessed at 12 months and 24 months [20].

### Program goals

The goals of the program were the following:

1. At least 7% of initial body weight loss at the end of the intervention program and maintenance of weight post-program;
2. Increase in PA (at least 150 minutes of moderate-intensity activity per week);
3. Promotion of healthy eating habits;
4. Decrease in weekly sedentary time;
5. Decrease of cardio-metabolic risks from baseline.

### Counseling sessions

The program, both in rural and urban study centers, started with an inaugural session addressing the participants of that area on different days (the rural program was started on the 1st Sunday of September, and the urban program started the following Sunday). The participants interacted with the study team, their health risks and future complications were discussed, and the importance of the program was delivered. Program-related leaflets and folders written in Bengali and activity logbooks were also distributed. All the participants in an area were divided into small groups based on their gender (15-16 in each group). A total of 16 individual or group sessions were arranged in each study center (rural & urban) for the first 24 weeks, and then it was limited to at least one session per month for each group. Each session usually lasted for 60-90 minutes. The topics were interdisciplinary and delivered by personnel trained in the respective field [21].

### Educational session

The educational session discussed obesity, its link with other NCDs, complications, prevalence and severity, economic burden, and possible treatment (lifestyle modification, medication, and surgery). The behavioral change includes prudential shopping of food, reading the food labels, or choosing the right foods [22].

### Dietary counseling

The principle of the individualized diet plans was a low-calorie, low-fat, and high-fiber diet that included a reduction of 500-1000 kcal/day. The total calorie distribution is likely to be 50-60% from carbohydrates, 15% from protein, 20%-30% from unsaturated fat, and 8-10% from saturated fat. Dietary salt was limited to 6 gm/day. The program advised the consumption of low glycemic index foods, an increase in fruit and vegetable (except potato) intake of up to 5 servings per day, the consumption of whole fruits instead of fruit juices and the replacement of carbonated beverages with milk. After attaining a desirable body weight, a maintenance diet was recommended. Participants were asked to record their daily intake, including portion size, in activity logbooks and were given leaflets with a list of foods along with their nutritional composition from a standard portion size (100gm) [23].

### Physical activity session

The participants were encouraged to increase moderate-intensity activity to at least 150 minutes a week, initially, and to increase the activity time gradually. They were advised to find their own way to accomplish the required activities. Two approaches were recommended, one was self-programmed exercise (e.g., brisk walking, bicycling), and the second was lifestyle exercise (e.g., using stairs instead of lifts, avoiding vehicles for short distances). In general, it was advised to walk for 30-45 minutes with more intensity 3-5 days/week and to reduce sedentary hours (e.g., screen time) to <2 hours/day. Participants were asked to record their activities, daily, in diaries, along with the time and intensity [24, 25]. The program also allotted a few sessions for encouragement, problem-solving, or motivational activities, so the participants could better adapt and adhere to the modified lifestyle.

### Outcome measures and timing of measurements

#### Primary outcomes

Data regarding anthropometric and clinical variables were measured at three levels (1) baseline (before intervention), (2) post-intervention (at 12 months), and (3) at follow-up (at 24 months). Anthropometric measurements, including body weight, height, BMI (kg/m^2^), WC (cm), WHR, WHtR were assessed at the respective study centers by using standard protocols and instruments [26, 27]. SBP (mmHg) and DBP (mmHg) were measured in a seated position, after at least 10 minutes of rest, by standard sphygmomanometer with adult cuffs. These measurements were taken twice at an interval of 3 minutes, and the mean was calculated and recorded [28]. Venous blood samples (5 ml) were drawn by expert lab technicians after 8-12 hours of overnight fasting for biochemical tests (e.g., FBG, fasting plasma insulin level, and lipid profile which includes TC, HDL-C and LDL-C, and TG). The blood collection, storage, and tests were done following standard protocols [29]. HOMA was used to evaluate insulin resistance (IR) by using the formula [HOMA-IR = fasting insulin (µIU/mL) X fasting glucose (mmol/L)/22.5] [30].

#### Secondary outcomes

The assessment of the self-reported PA level and dietary habits was done through a standardized questionnaire and multiple non-consecutive 24-hours recall methods, to evaluate the impact of the intervention program [31-33].

### Statistical analysis

Data were analyzed using descriptive statistics like mean and standard deviation (SD); the difference between two and more groups was assessed by independent t-test and one-way ANOVA, respectively. The chi-square test was used for categorical data. The results were considered significant at p-value ≤0.05. All data were analyzed using SPSS version 2017 (IBM, USA).

## RESULTS

After screening 300 obese people, 121 subjects were selected from similar socio-demographic backgrounds. Their socio-demographic profile is shown in Table 1.

**Table 1:** Sociodemographic characteristics of participants (n=121).

Characteristics	Rural n=61 (%)	Urban n=60 (%)	P-value
**Gender**			
**Male**	30 (49.2)	30 (50)	N.S.**
**Female**	31 (50.8)	30 (50)	
**Age (mean±SD)**	34.3±10.5	34.5±7.8	N.S.*
**Marital status**			
**Not married**	13 (19.7)	15 (25)	N.S.**
**Married**	48 (80.3)	45 (75)	
**Occupation**			
**Professional/skilled worker**	8 (13.1)	16 (26.7)	N.S.**
**Self-employed**	6 (9.8)	9 (15)	
**Semi professional**	16 (26.3)	9 (15)	
**Housewives**	21 (34.4)	17 (28.3)	
**Students/unemployed**	10 (16.4)	9 (15)	
**Education**			
**8–12 grades**	23 (37.7)	16 (26.7)	N.S.**
**University or more**	38 (62.3)	44 (73.3)	
**Monthly income (Rs)**			
**>25000**	48 (78.7)	54 (90)	N.S.**
**<25000**	13 (21.3)	6 (10)	

* – independent t-test; ** – chi-square test; N.S. – non-significant differences.

No significant differences were found between rural and urban groups regarding the socio-demographic profile. The mean age was 34.3 years for the rural and 34.5 years for the urban group. Professionally, all were sedentary workers.

No significant differences between the groups (rural and urban) were found regarding dietary and PA profiles (Table 2).

**Table 2:** Baseline dietary and physical activity profiles of participants (n=121).

Baseline profiles	Rural male (n=30)	Rural female (n=31)	Rural total (n=61)	Urban male (n=30)	Urban female (n=30)	Urban total (n=60)	Statistical significance
**Calorie (kcal)**	2402.09±170.05	1921.1±125	2130±376.6	2462.4±179.2	2019.2±173	2211.1±384	N.S.
**Sitting Time/Screen Time (minutes)**	335.4±66.9	357.09±55.5	346.2±61.3	355.7±79.8	368.9±52.3	368.1±64.9	N.S.
**MET Score/Week**	542.13±82.4	522.8±89.2	532.5±87.3	535.06±87.3	489.7±123.2	512.4±108.3	N.S.

Independent t-test; N.S. – non-significant differences.

Result of Primary outcomes included:

1. Changes in the anthropometric profile of subjects at baseline (prior-intervention), at 12 months (post-intervention), and 24 months (follow-up), represented in Table 3.
2. Changes in cardio-metabolic risks (blood pressure, FBG, plasma insulin, HOMA-IR, lipid profile) from baseline to follow-up, presented in Table 4 A, B.

**Table 3:** Changes in anthropometric parameters between rural and urban participants from baseline to follow-up (n=121).

Outcomes	Male (n=60)			Female (n=61)		
Anthropometric	Rural (30) mean±SD	Urban (30) mean±SD	P-value	Rural (31) mean±SD	Urban (30) mean±SD	P-value
**Bodyweight (kg)** **Baseline**	79.57±5.77	83.63±4.68	0.004	71.16±4.42	72.39±4.88	N.S.
**Post-intervention**	74.42±6.18	78.46±4.67	0.006	65.64±4.25	67±4.66	N.S.
**Follow-up**	75.6±6.6	79.9±5.12	0.007	66.6±4.5	67.98±4.84	N.S.
**F value**	2.92	4.95		7.006	5.612	
P-value**	0.015	0.001		0.001	0.001	
**BMI (kg/m^2^)** **Baseline**	27.2±1.11	27.9±1.23	0.02	27.95±1.28	28.11±1.19	N.S.
**Post-intervention**	25.43±1.3	26.2±1.27	0.02	25.8±1.3	26.02±1.18	N.S.
**Follow-up**	25.84±1.47	26.68±1.52	0.03	26.15±1.48	26.4±1.37	N.S.
**F-value**	8.33	7.37		11.34	13.84	
P-value**	0.001	0.001		0.001	0.001	
**WC (cm)** **Baseline**	99.33±3.12	101.07±2.66	0.024	93.81±2.42	94±2.88	N.S.
**Post-intervention**	97.3±3.01	99.3±2.9	0.01	90.7±3.08	91.7±2.64	N.S.
**Follow-up**	98.5±3.02	100.5±2.91	0.01	92.85±2.38	93.37±2.62	N.S.
**F-value**	3.31	3.04		11.5	5.5	
P-value**	0.04	0.05		<0.0001	0.006	
**WHR** **Baseline**	0.988±0.026	0.998±0.017	N.S.	0.89±0.017	0.9±0.015	N.S.
**Post-intervention**	0.967±0.024	0.977±0.02	N.S.	0.87±0.015	0.88±0.013	0.002
**Follow-up**	0.98±0.024	0.992± 0.02	N.S.	0.88±0.017	0.89±0.012	N.S.
**F-value**	5.47	9.43		15.04	14.74	
P-value**	0.005	0.0002		<0.0001	<0.0001	
**WHtR** **Baseline**	0.578±0.015	0.581±0.015	N.S.	0.583±0.01	0.582±0.015	N.S.
**Post-intervention**	0.564±0.01	0.568±0.014	N.S.	0.57±0.012	0.571±0.016	N.S.
**Follow-up**	0.574±0.016	0.578±0.018	N.S.	0.580±0.011	0.580±0.013	N.S.
**F-value**	6.59	4.87		9.05	4.57	
P-value**	0.002	0.009		0.0003	0.012	

** – ANOVA and * – t-test. F-value: the degree of variation between the means of two or more groups. N.S. – non-significant (p>0.05).

**Table 4:** Change in primary outcomes among rural and urban subjects from baseline to follow-up (n=121).

A. Bio-chemical changes						
Outcomes	Males (n=60)			Females (n=61)		
Biochemical	Rural (30) mean±SD	Urban (30) mean±SD	P-level	Rural (31) mean±SD	Urban (30) mean±SD	P-level
**FBG (mg/dl)** **Baseline**	110.03±8.88	110±9.86	N.S.	106.84±8.77	107.03±8.77	N.S.
**Post-intervention**	98.93±7.6	97.9±6.82	N.S.	98.22±7.7	95.6±6.21	N.S.
**Follow-up**	98.8±6.81	97.36±8.06	N.S.	97.06±6.5	97.63±6.89	N.S.
**F-value**	20.38	21.98		14.8	20.53	
P-value**	0.001	0.001		0.001	0.001	
**Plasma Insulin (microU/L)** **Baseline**	11.21±3.48	13.25±4.4	N.S.	9.93±1.34	13.14±3.71	0.001
**Post-intervention**	10.71±3.43	12.77±4.17	0.04	9.52±1.16	12.75±3.61	0.001
**Follow-up**	10.79±3.47	12.83±3.98	0.04	9.7±1.21	12.9±3.5	0.001
**F-value**	0.17	0.116		0.845	0.88	
P-value**	N.S.	N.S.		N.S.	N.S.	
**HOMA-IR** **Baseline**	3.07±1.16	3.65±1.44	N.S.	2.64±0.48	3.49±1.19	0.001
**Post-intervention**	2.65±1.06	3.1±1.1	N.S.	2.3±0.37	3.02±0.97	0.001
**Follow-up**	2.66±1.09	3.11±1.14	N.S.	2.32±0.37	3.11±0.91	0.001
**F-value**	1.41	1.9		6.52	1.752	
P-value**	N.S.	N.S.		0.002	N.S.	
**TC (mg/dl)** **Baseline**	228±36.34	249.82±34.71	0.02	227.32±32.25	238.5±38.21	N.S.
**Post-intervention**	210.1±35.31	232.12±29.11	0.01	211.29±32.22	220.7±38.54	N.S.
**Follow-up**	211.7±34.7	235.22±29.68	0.006	213.39±32.95	228.13±31.27	N.S.
**F-value**	2.34	2.73		2.32	1.83	
P-value**	N.S.	N.S.		N.S.	N.S.	
**HDL-C (mg/dl)** **Baseline**	40.2±3.18	39.86±2.56	N.S.	40.51±1.89	41.16±2.29	N.S.
**Post-intervention**	40.3±3.04	40.3±2.15	N.S.	40.74±2.29	41.6±2.23	N.S.
**Follow-up**	40.3±3.24	40.06±2.18	N.S.	40.26±2.25	41.03±2.37	N.S.
**F-value**	0.014	0.26		0.39	0.49	
P-value**	N.S.	N.S.		N.S.	N.S.	
**TG (mg/dl)** **Baseline**	150.93±26.62	169.07±23.3	0.007	142.04±25.96	158.77±25.09	0.01
**Post-intervention**	145.19±39.81	155.77±20.3	N.S.	131.9±24.1	144.43±22.32	0.04
**Follow-up**	140.1±23.85	157.9±22.7	0.004	134.8±19.03	150.63±19	0.04
**F-value**	0.92	3.1		1.8	3.12	
P-value**	N.S.	0.05		N.S.	0.04	
**LDL-C** **Baseline**	132.3±9.86	140.96±11.27	0.002	131.06±8.15	134.9±10.07	N.S.
**Post-intervention**	131.3±9.93	140.13±11.66	0.003	131.19±8.13	133.3±9.64	N.S.
**Follow-up**	132±9.81	141.23±11.32	0.001	132.42±7.77	135.4±9.71	N.S.
**F-value**	0.75	0.075		0.269	0.375	
P-value**	N.S.	N.S.		N.S.	N.S.	
**B. Changes in blood pressure**						
**Outcomes**	**Males (n=60)**			**Females (n=61)**		
**Blood Pressure (B.P)**	**Rural (30) mean±SD**	**Urban (30) mean±SD**	p≤0.05 (between both groups) *	**Rural (31) mean±SD**	**Urban (30) mean±SD**	p≤0.05 (between both groups) *
**SBP (mmHg)** **Baseline**	132.23±4.81	133.5±4.55	N.S.	132.32±5.5	132.57±4.56	N.S.
**Post-intervention**	131.46±4.93	132.16±4.44	N.S.	131.71±6.5	131.46±4.59	N.S.
**Follow-up**	132.86±3.72	133.93±4.96	N.S.	134.39±6.4	133.8±4.81	N.S.
**F-value**	0.72	1.16		1.052	1.08	
P-value**	N.S.	N.S.		N.S.	N.S.	
**DBP (mmHg)** **Baseline**	80.96±2.78	82.7±2.47	0.01	81.06±3.28	80.4±2.94	N.S.
**Post-intervention**	80.93±3.11	81.86±3.35	N.S.	81.09±3.6	80.4±2.47	N.S.
**Follow-up**	81.73±3.53	82.9±2,55	N.S.	82.9±4.06	81.5±3.27	N.S.
**F-value**	0.61	1.13		1.404	0.8	
P-value**	N.S.	N.S.		N.S.	N.S.	

** – ANOVA and * – t-test. F-value: the degree of variation between the means of two or more groups. N.S. – non-significant (p>0.05).

Among rural subjects, 60.7% attained 7% weight loss from the initial values, whereas in the urban setting, the percentage of subjects was 61.7%. However, this difference was statistically insignificant (p=0.9**, chi-square test). All anthropometric parameters changed significantly across all groups from baseline.

Among all clinical parameters, FBG significantly decreased among all groups over time. HOMA-IR declined in the rural female group, and the TG level changed significantly among urban participants. No other significant changes were observed.

Secondary outcomes included a change in food behaviour and physical activity levels after intervention and adherence to this modified behaviour during the follow-up period, i.e., after 24 months (Table 5 A, B).

**Table 5:** Change in secondary outcomes among rural and urban subjects from baseline to follow-up (n=121).

A. Dietary pattern				
Outcomes	Males (n=60)		Females (n=61)	
Dietary profile	Rural (30) mean±SD	Urban (30) mean±SD	Rural (31) mean±SD	Urban (30) mean±SD
**Calorie (kcal)** **Baseline**	2402.09±170.05	2462.4±179.2	1921.1±125	2019.2±173
**Post-intervention**	2006.17±99.82	2010.7±118.85	1806.9±103.8	1824.3±113.21
**Follow-up**	2064.17±134.98	2083.7±130.37	1888.84±119.33	1900.33±98.78
**F-value**	72.06	83.64	7.64	16.5
P-value**	<0.0005	<0.0005	0.0008	<0.0005
**Cereals (whole & refined) g** **Baseline**	309.63±38.1	301.6±22.7	294.04±26.3	301.8±14.6
**Post-intervention**	270.4±37.31	260.2±59.12	237.13±52.2	243.03±42.7
**Follow-up**	277.5±29.8	269.9±57.16	239.6±52.05	246.5±49.8
**F-value**	10.52	5.79	15.24	21.6
P-value**	<0.0005	0.004	<0.0005	<0.0005
**Milk & products (ml)** **Baseline**	91.9±18.9	88.06±12.7	65.8±24.5	84.1±23.8
**Post-intervention**	111.4±30.4	98.6±19.3	110.9±46.41	107.9±30.27
**Follow-up**	105.73±23.96	102.7±21.74	103.32±36.95	98.33±29.27
**F-value**	4.87	5.11	13.13	4.38
P-value**	0.009	0.007	<0.0005	0.015
**Vegetables (except potato) g** **Baseline**	92.3±19.4	91.4±21.7	82.6±17.1	73.8±19.9
**Post-intervention**	114.6±31.7	107.6±24.7	111.42±19.6	102.9±17.35
**Follow-up**	107.5±21.5	107.7±8	112.3±19.1	101.9±11.5
**F-value**	6.33	6.92	25.6	29.7
P-value**	0.002	0.0016	<0.0005	<0.0005
**Fruits (g)** **Baseline**	40.6±10.5	45.5±9.6	40.61±10.6	47.3±27.6
**Post-intervention**	95.4±24.2	98.9±25.13	92.2±29.7	102.4±30.7
**Follow-up**	90.6±30.5	88.9±23	86.3±26.5	91.4±26.5
**F-value**	51.14	57.9	43.58	34.9
P-value**	<0.0005	<0.0005	<0.0005	<0.0005
**Visible fat/oils (g)** **Baseline**	39.4±8.5	43.4±9	40.6±10.6	41.8±8.1
**Post-intervention**	38.9±9.15	41.6±6.7	38.3±8.7	39.3±5.3
**Follow-up**	40.53±11.4	42.9±6.8	39.2±7.7	41.4±5.8
**F-value**	0.22	0.43	0.53	1.31
P-value**	N.S.	N.S.	N.S.	N.S.
**Added sugar (g)** **Baseline**	31.8±5.9	34.8±9	34.83±8.8	34.5±6
**Post-intervention**	31.3±4.5	33.8±4.8	33.8±4.8	33.23±6.34
**Follow-up**	31.57±4.42	34.53±5.4	34.53±5.4	34.2±6.04
**F-value**	0.087	0.19	0.015	0.35
P-value**	N.S.	N.S.	N.S.	N.S.
**B. PA pattern**				
**Outcomes**	**Males (60)**		**Females (61)**	
**PA profile**	**Rural (30) mean±SD**	**Urban (30) mean±SD**	**Rural (31) mean±SD**	**Urban (30) mean±SD**
**Sitting time/ day (minutes)** **Baseline**	335.4±67	357.09±55.5	355.7±79.8	368.9±52.3
**Post-intervention**	235.43±60.8	237.9±79.6	262.8±68.9	301.2±40
**Follow-up**	262.5±64.03	263.8±77.2	284.2±60	317.8±48
**F-value**	19.6	23.8	14.5	16.9
P-value**	<0.0005	<0.0005	<0.0005	<0.0005
**MET score/week** **Baseline**	542.13±82.4	522.9±89.17	535.06±87.29	489.7±123
**Post-intervention**	684.53±134.3	681.9±140.9	702.06±148.4	690.9±155
**Follow-up**	652.2±154	646.7±110.3	671.26±139.05	633.3±174
**F-value**	10.3	16.2	14.5	13.9
P-value**	<0.0005	<0.0005	<0.0005	<0.0005

** – ANOVA. F-value: the degree of variation between the means of two or more groups. N.S. – non-significant (p>0.05).

Except for the consumption of fats, oils, and added sugar, all other dietary and PA variables changed significantly among all groups.

## DISCUSSION

This lifestyle intervention study for the management of obesity and reduction of cardio-metabolic complications of rural and urban obese subjects of both sexes is the first ever attempt in West Bengal, as per our knowledge. In this research, we aimed to explore the effect of lifestyle intervention programs through a change in dietary patterns and PA profiles of participants with similar socio-demographic backgrounds in the Hooghly district of West Bengal. Lifestyle modification brought significant changes among all anthropometric parameters and FBG (mg/dl) levels across all four groups (rural male, urban male, rural female, and urban female) from baseline to follow-up. A significant reduction has been noticed in serum TG (mg/dl) levels among urban obese participants (both male and female groups), and insulin sensitivity improved among rural women only. A between-group comparison (rural male vs. urban male; rural female vs. urban female), indicated significant differences regarding serum TG (mg/dl) and DBP (mmHg) at baseline between the two groups, which became insignificant after intervention. However, during follow-up, TG (mg/dl) differences between groups became significant, though DBP (mmHg) remained unchanged.

The secondary outcome, the adaptation and adherence to a modified lifestyle, showed a remarkably significant decrease with respect to calorie and cereal consumption; notably, consumption of milk beverages and products, fruits, and vegetables (except potato) showed excellent improvements. No significant change was found regarding oil or fat and added sugar intake among all four groups over time. Sitting time (minutes) declined significantly from baseline to post-intervention; though the mean value increased during the follow-up period. Mean values of MET-score among all groups indicated that subjects kept active even during the follow-up period, as per the recommendation of WHO to prevent NCDs.

This interventional study presented a significant effect in reducing the cardio-metabolic complications of obese participants of both sexes from rural and urban areas. Obesity is the most common outcome of a modern, fast-paced lifestyle which dysregulates inflammatory markers, resulting in several metabolic or cardiovascular disorders. Insulin resistance, diabetes, hypertension, dyslipidemia, metabolic syndrome, are among the long-term consequences of obesity. Studies proved that prevention or treatment of obesity could effectively lessen the cardio-metabolic risks [34, 35]. Dietary management or improvement of PA level alone could not produce a better result than their combined effect in preventing obesity. Therefore, lifestyle intervention through a combination of dietary modification and improved PA among target populations showed a positive impact on tackling various health issues related to obesity (e.g., diabetes, insulin resistance, cardio-metabolic or liver abnormalities, and osteoarthritis) [36].

Many interventional studies on obesity or related complications used lifestyle modification as a potential tool to shed extra weight, produce a better insulin response, revert pre-hyperglycaemic to normoglycemic, and proved to improve lipid or other cardio-metabolic profiles [37]. Several randomized controlled trials (RCT) or longitudinal studies were carried in South Asia to prevent obesity among children and adults over decades. The meta-analysis by Brown et al. 2015 outlined that the outcomes of such intervention studies varied [38]. One RCT program designed for 3 years, based on a calorie deficit diet and brisk walking for 30 minutes daily, produced a significant reduction in body weight, BMI, or WC in the intervention group, whereas other research did not showcase such prominent effects. This could be partly explained by the variation in study design [38-40]. Chapman et al. 2013 reviewed that diet and PA have significantly improved anthropometric parameters, but no effect was found regarding insulin sensitivity, though few studies noted the change in DBP or lipid profile after the intervention. In this study, male participants showed better weight loss than female participants. However, the subjects were from different religions, which affected the results due to the fact that the physical activity sessions included both genders. As a result, many women might have been unwilling to attend, influencing the result [41]. In our study, we targeted the same religious and cultural group, and counseling or activity sessions were scheduled separately for both sex groups, revealing that female obese participants presented better results than their male counterparts, as they attended more counseling sessions and showcased better adherence to modified lifestyles. This observation was similar to the rural-urban study done in Tamilnadu, India, in 2011, which noted that women were more cooperative than men, leading to better outcomes [42]. Sex difference became insignificant where attendance and completion of counseling sessions was the independent factor in the weight loss program [43].

In our study, we used a calorie, carbohydrate, and saturated fat-restricted diet with plenty of fibers, along with moderate PA, to reduce body fat and to improve other cardio-metabolic profiles. 60.7% of rural and 61.7% of urban obese participants achieved 7% of the initial body weight loss goal at 12 months. The mean change in body weight ranged from -5.1 kg to -5.6 kg; BMI -1.7 to -2.08 kg/m^2^; WC -1.8 to -3.1 cm across all four groups after intervention. This finding goes in line with observations noted in other research on various populations [36, 42, 44-46]. Research in Kerala showed that at 24 months subjects who received intervention did not produce better results, with little loss in body weight, WC, non-significant change in fruit intake, and sedentary behavior. This may be due to a difference in study criteria and subject selection, which was based on IDRS (Indian Diabetes Risk Score) but not obesity, so subjects with lower body weight or WC with high IDRS were included [13].

The cardio-vascular and biochemical results were comparable with other studies like Backes et al. 2008, where carbohydrate and calorie-restricted diet reduced body weight, FBG, TG, and DBP but showcased no significant difference in TC, HDL-C, and LDL-C [47]. IDPP-1 (Indian diabetic prevention Program) showed insignificant change in the lifestyle modified intervention group regarding body weight or WC, but improved insulin sensitivity, which may be due to the lower BMI of subjects at baseline, and adapting dietary modification and increased PA might have reduced metabolic risk among those subjects [48]. Nanditah et al. (2014) showed men on lifestyle modification for 24 months improved insulin response and the observed obesity was associated with dysglycemia, which again related to cardio-metabolic risks [49]. A 12-month lifestyle intervention program in Germany in 2018 also showed a beneficial result for obese subjects, as losing weight led to a better cardio-metabolic profile [9]. Weight loss through lifestyle modification has also been proven to improve liver function [50]. Yadav et al. (2018) showed that yoga-based lifestyle modification has a better result than dietary intervention alone on inflammatory markers of metabolic syndrome [51]. In our study, participants lost a significant portion of body weight or abdominal fat but failed to influence biochemical parameters except for blood glucose and TG level, which indicate the underlying metabolic complications, genetic influence, body composition, or requirement of more fat to lose [52].

Secondary outcomes showed subjects adopted healthier food habits, but it was not as per recommendations by ICMR-NIN (Indian Council of Medical Research–National Institute of Nutrition). The consumption of calories and cereals have declined, and fruit, vegetables (except potato), and milk intake have raised significantly, yet no significant difference was found regarding consumption of visible fat or added sugar among all groups. PA level has raised, and all groups became active (MET-score >600 MET) even during the follow-up. Sedentary time has fallen among all groups significantly as well. Though the mean values of MET score and sedentary time have declined from post-intervention values, they did not cross the baseline in any group. Other interventional studies have also identified improved dietary habits and PA profiles adopted by a good proportion of the target group and succeeded in attaining their study goal. K-DPP also showed improvement in dietary habits and PA behavior among 99% and 96% of participants, respectively [53]. Mathews et al. (2021) in their study on the effectiveness of PA among sedentary women in Thiruvanthapuram, India, noted that women preferred moderate-intensity activity like walking or household work, and MET minute increased significantly from baseline with a simultaneous reduction in WC, similarly to our results [54].

The Diabetes Community Lifestyle Improvement Program (D-CLIP) trial in Chennai, India, also showed improvement in Moderate to Vigorous Physical Activity (MPVA) among intervention groups in diabetes prevention programs in India [55]. Another study noted lifestyle management is more effective than drug treatment to prevent diabetes, and 450±26 kcal energy intake was reduced by the intervention group; fat intake was decreased, and 74% adhered with the recommendation of >150 minutes MVPA and resulting in average weight loss of 5.6 kg (mean value) and succeeded to improve diabetes. Vadheim et al. 2010 in a lifestyle intervention program to lower cardio-metabolic risks in the rural community, reported that 52% of participants who received the intervention achieved a 7% weight loss goal after 16 weekly sessions, and 65% of them met the PA goal [56]. Other interventional research also showed an increase in MET score, fruit and vegetable consumption, and successive weight loss. The mean values of primary or secondary outcomes started to increase during follow-up, as supported by other studies [24, 34, 57].

### Limitations

This study has certain limitations, like a high attrition rate (28 out of 149 subjects, i.e., 18.8%), gradually lower attendance in counseling sessions, and declined responses after the first 6 months, which may indicate that the program was not attractive or motivating. Self-reported dietary and activity log books for dietary and PA assessment could be biased, and the findings cannot be generalized due to a small sample size.

## CONCLUSION

The lifestyle intervention program is of utmost importance in the present society to prevent obesity and its deleterious consequences among all age groups worldwide. Such intervention programs can enhance mass awareness regarding healthy living as the cheapest way to prevent NCDs. Lifestyle intervention programs can be applied in educational settings or workplaces. Government initiatives and other stakeholders should come forward to support resources like trained health personnel, funds, or accommodation of community buildings to conduct such programs more, which not only lessen the economic burden for diseases of the countries but also get healthy active posterity.

## Special thanks

The authors convey their heartfelt thanks to Mr. HemantaRouth, senior dietitian of the Department of Nutrition and Dietetics in IQ City Medical College, Burdwan, West Bengal, for his relentless support and cooperation in this program.
